# Cholinesterase inhibitors, donepezil and rivastigmine, attenuate spatial memory and cognitive flexibility impairment induced by acute ethanol in the Barnes maze task in rats

**DOI:** 10.1007/s00210-016-1269-8

**Published:** 2016-07-04

**Authors:** Kinga Gawel, Krzysztof Labuz, Ewa Gibula-Bruzda, Malgorzata Jenda, Marta Marszalek-Grabska, Joanna Filarowska, Jerzy Silberring, Jolanta H. Kotlinska

**Affiliations:** 1 Department of Pharmacology and Pharmacodynamics, Medical University, Chodzki 4A, 20-093 Lublin, Poland; 2 Sahlgrenska University Hospital, 413 45 Göteborg, Sweden; 3 Department of Biochemistry and Neurobiology, AGH University of Science and Technology, Mickiewicza 30, 30-059 Krakow, Poland

**Keywords:** Barnes maze, Spatial memory, Ethanol, Donepezil, Rivastigmine

## Abstract

Central cholinergic dysfunction contributes to acute spatial memory deficits produced by ethanol administration. Donepezil and rivastigmine elevate acetylcholine levels in the synaptic cleft through the inhibition of cholinesterases—enzymes involved in acetylcholine degradation. The aim of our study was to reveal whether donepezil (acetylcholinesterase inhibitor) and rivastigmine (also butyrylcholinesterase inhibitor) attenuate spatial memory impairment as induced by acute ethanol administration in the Barnes maze task (primary latency and number of errors in finding the escape box) in rats. Additionally, we compared the influence of these drugs on ethanol-disturbed memory. In the first experiment, the dose of ethanol (1.75 g/kg, i.p.) was selected that impaired spatial memory, but did not induce motor impairment. Next, we studied the influence of donepezil (1 and 3 mg/kg, i.p.), as well as rivastigmine (0.5 and 1 mg/kg, i.p.), given either before the probe trial or the reversal learning on ethanol-induced memory impairment. Our study demonstrated that these drugs, when given before the probe trial, were equally effective in attenuating ethanol-induced impairment in both test situations, whereas rivastigmine, at both doses (0.5 and 1 mg/kg, i.p.), and donepezil only at a higher dose (3 mg/kg, i.p.) given prior the reversal learning, attenuated the ethanol-induced impairment in cognitive flexibility. Thus, rivastigmine appears to exert more beneficial effect than donepezil in reversing ethanol-induced cognitive impairments—probably due to its wider spectrum of activity. In conclusion, the ethanol-induced spatial memory impairment may be attenuated by pharmacological manipulation of central cholinergic neurotransmission.

## Introduction

Ethanol is one of the most frequently abused drugs in our society, and there is a widespread agreement that acute ethanol intoxication affects memory and attention in humans (Goodwin et al. 1970; Tharp et al. 1974; Weissenborn and Duka 2000). In laboratory animals, acute intraperitoneal ethanol administration selectively impairs the spatial memory (Berry and Matthews 2004; Matthews et al. 1995; Matthews et al. 1999) which is necessary to accurately navigate within the environment (Brazhnik et al. 2003; Mehta 2015). It has been demonstrated that ethanol and hippocampal system damage produce similar patterns of learning and memory impairment (Matthews et al. 1996; Ryabinin 1998).

Cholinergic projections of the basal forebrain/nucleus *basalis magnocellularis* and *medial septum* (basal forebrain cholinergic complex) to the cerebral cortex and hippocampus have long been regarded as critical for memory (Bartus et al. 1985; Schliebs and Arendt 2011; Teles-Grilo Ruivo and Mellor 2013). Some studies have reported that the reduction in hippocampal acetylcholine (ACh) levels (either natural due to aging or pharmacological) specifically correlates with impairment in spatial memory (Ikegami 1994; Mishima et al. 2000; Gold 2003). Interestingly, acute systemic ethanol exerts a biphasic effect on ACh release in the hippocampus: apparently, low doses (0.8 g/kg) increase hippocampal ACh release, by a direct effect on the septohippocampal pathway, while a sedative dose (2.4 g/kg) reduces ACh release (Henn et al. 1998). This inhibitory effect may be responsible for the well-known cognitive effects of acute ethanol administration, such as learning impairment and amnesia. However, correlation between the behavioral effects of low ethanol dose and hippocampal ACh release is less understood (Stancampiano et al. 2004).

Another brain region that receives cholinergic projection from the nucleus basalis magnocellularis is the prefrontal cortex (PFC) (Mesulam et al. 1983). Several studies have demonstrated that interactions between the PFC and hippocampus are involved in spatial memory (Lee and Kesner 2003; Kyd and Bilkey 2003; Wang and Cai 2006). However, the PFC incorporates several distinct areas of the frontal cortex that are associated with processes involved in executive functions, including working memory, attention, cognitive flexibility, and impulse control (Logue and Gould 2014). These complex behaviors allow for adaptation in response to changes in the environment and are modulated by various neurotransmitters, including the cholinergic system (Logue and Gould 2014). Acute ethanol injection shows a dose-dependent effect on ACh release in PFC (Stancampiano et al. 2004): low intraperitoneal doses of ethanol (0.5 g/kg) increase, while higher doses (1 g/kg) reduce ACh release in the rat PFC (Stancampiano et al. 2004; Jamal et al. 2010). These biphasic changes of ACh transmission in the PFC may be of relevance for the bidirectional modulation of working memory by ethanol (Rossetti et al. 2002; Stancampiano et al. 2004).

In the mammalian brain, synaptic levels of ACh are regulated by two types of cholinesterases: acetylcholinesterase and butyrylcholinesterase that inactivate ACh (Giacobini 2004; Ballard et al. 2005). Treatment with cholinesterase inhibitors increases ACh level. Donepezil is a centrally acting, reversible acetylcholinesterase inhibitor (Dooley and Lamb 2000; Liston et al. 2004), while rivastigmine is a highly potent agent that blocks both acetylcholinesterase and butyrylcholinesterase (Ogura et al. 2000; Racchi et al. 2004). These drugs are approved by the US Food and Drug Administration (FDA) as a first-choice therapy for the treatment of mild to moderate Alzheimer’s disease (Zemek et al. 2014).) It should be noted that disturbances in the cholinergic system as well as spatial memory impairments are observed in Alzheimer’s disease and after chronic ethanol treatment (Adelstein et al 1992**;** Arendt et al. 1988, b**;** Bartus et al. 1982; Monacelli et al. 2003).

The aim of the present study was to examine whether the cholinesterase inhibitors, donepezil and rivastigmine, are capable of modifying short-term spatial memory and cognitive flexibility (reversal learning) impairments caused by acute ethanol administration in the Barnes maze task in rats. The task is based on the assumption that the animal should learn and remember the location of a safe shelter (Carrillo-Mora et al. 2009), and the test consists of several phases. These include a habituation phase, in which the animals are introduced to the environment and task, and the acquisition phase (training), during which the animals learn to find the location of the shelter. These are followed by a test consisting of two parts. The first part of the test (called the “probe trial”) is carried out with the unchanged position of the shelter in relation to the acquisition sessions—this allows an assessment of spatial memory retention, which is a subset of the short-term memory (24 h after the training sessions) required to perform certain operations (Carrillo-Mora et al. 2009; Cowan 2008; Brickman and Stern 2009). In turn, the second part of the test (called “reversal learning”), by changing the target position, allows for the assessment of cognitive flexibility necessary to relearn a new location that is no longer rewarded (Carrillo-Mora et al. 2009). The advantage of the Barnes maze task as compared to other animal models is the elimination of highly stressful stimulus (e.g., water).

Furthermore, in the current experiments, we compared the influence of donepezil, which functions solely as a pharmacological inhibitor of acetylcholinesterase, and rivastigmine, which also acts as a butyrylcholinesterase inhibitor (Dooley and Lamb 2000; Liston et al. 2004; Ogura et al. 2000; Racchi et al. 2004), on acute ethanol-induced impairment in the spatial memory and cognitive flexibility in the Barnes maze task. In the first experiment, the dose of ethanol that impaired spatial memory, but did not induce motor impairment, was selected. Such dose was used in further studies. In our study, we expected stronger beneficial effects of rivastigmine on the improvement of ethanol-induced memory deficiency, due to a wider spectrum of activity, as compared to donepezil.

## Materials and methods

### Animals and drugs

Male Wistar rats (HZL, Warsaw, Poland), weighing 200–250 g at the beginning of the experiment, were habituated for at least 1 week before the experiment. The animals were maintained under the standard laboratory conditions (22 °C, 12:12 light/dark cycle) and housed ten per cage with food (Agropol, Motycz, Poland) and water available ad libitum. A total of 292 animals were used. All behavioral studies were performed between 09:00 and 17:00. All experimental protocols and housing conditions were approved by the Local Ethics Committee and carried out according to the National Institute of Health Guidelines for the Care and Use of Laboratory Animals and the European Community Council Directive of November 2010 for Care and Use of Laboratory Animals (Directive 2010/63/EU) and were approved by the Local Ethics Committee.

The following drugs were used: ethanol (95 %, POCH, Gliwice, Poland) was mixed with 0.9 % NaCl (saline) to make 10 % *w*/*v* solution and was administered by intraperitoneal (i.p.) injection. Donepezil hydrochloride (Sigma-Aldrich, St. Louis, MO, USA) and rivastigmine (Sigma-Aldrich, St. Louis, MO, USA) were dissolved in saline and injected in a volume of 2 ml/kg, i.p. Control groups received saline injections in the same volume and by the same route.

### Barnes maze task

The Barnes maze (Stoelting, Dublin, Ireland) consisted of a gray metal, circular platform of 122 cm diameter, elevated 90 cm above the floor, with 20 equally spaced holes (10 cm diameter) located in the periphery. One of the holes was connected to an escape box of 35 cm × 12 cm × 12 cm, of the same material and color as the platform. The other holes were covered underneath with a flat box, also of the same material and color. From the center of the maze, all holes looked identical, so that the rats could not discriminate the escape hole from other holes until situated adjacent to it. Numerous visual cues (in the form of large colorful geometric shapes) were placed on the walls of the testing room at 1–2 m distance from the edge of the maze. To further evoke the potentiated escape response, the platform was brightly lit (two points of light 1.5 m above the maze; 500 W each) and a buzzer placed above the center of the maze provided a sound of 80 dB as an additional aversive stimulus to provoke the escape from the platform.

The Barnes maze task was carried out according to the method described by Kuzmin et al. (2012) with minor modifications. The Barnes maze task consisted of the following phases: (i) adaptation phase (habituation); (ii) acquisition phase; and (iii) test phase (probe trial and reversal learning).

#### *Habituation*

One day before the acquisition phase, the rats were habituated to the platform and the escape box to reduce anxiety behavior. This habituation trial was performed with the lights on, but without the buzzer sound.

#### *Acquisition phase*

The acquisition phase began 24 h after the maze habituation. Acquisition involved one training session per day for 4 consecutive days. Each training session consisted of three 180-s trials, with 10-min inter-trial interval during which animals returned to their home cage. The location of the platform and the escape box remained constant over all the acquisition trials. Each trial began by placing the animal at the center of the platform, the buzzer was excited, and rats were allowed to freely explore the apparatus. The trial was completed after 180 s or when the animal entered the escape box. Immediately after entering the escape box, the buzzer was turned off and the hole was covered for 30 s before the rat was returned to home cage. If the animal did not enter the goal box within 180 s, it was gently guided there by the experimenter and could explore it for 30 s. To eliminate olfactory cues, the platform surface and the goal box were wiped with a 10 % (*w*/*v*) ethanol solution after each trial to dissipate odor cues and provide a standard olfactory context for each trial. All trials were recorded by a trained observer. Since the animals occasionally lacked motivation and merely explored the maze after finding the escape box without entering into it, following the work of many authors (Harrison et al. 2006; Li et al. 2014; Patil et al. 2009), in our experiments, we scored such parameters as the primary latency and primary errors. Primary latency was defined as the time required for the rat to make initial contact with the escape box. Primary errors were defined as the number of holes visited before the first contact with the escape box.

#### *Probe trial*

One day after the acquisition phase (i.e., on day 5), the subjects received a probe trial for 90 s to evaluate spatial memory. During this trial, the tunnel leading to the escape box was closed (Li et al. 2014; Patil et al. 2009). The rats were allowed to explore the maze and investigate the escape box and the adjacent holes. The primary latency and primary errors to reach the escape box were counted.

#### *Reversal learning*

One hour after completion of the probe trial, three 180-s reversal learning trials were conducted. Reversal learning trials were identical to the acquisition trials, except that the position of the escape hole was rotated 180°. The rat was, therefore, unable to escape the maze using the acquired spatial cues, but had to relearn the new location of the hole. Data obtained from the reversal learning trials were pooled together and used for calculations of primary latency and primary errors.

### Locomotor activity

The locomotor activity of individual rats was recorded using a photocell apparatus (Porfex, Bialystok, Poland). The animals were individually placed in Plexiglas boxes (square cages, 60 cm a side) in a sound-attenuated experimental room. The cages were equipped with two rows of infrared, light-sensitive photocells, located 40 and 100 mm above the floor. Locomotor activity was recorded as a horizontal activity (distance traveled) by each rat for a total period of 15 min.

### Rotarod performance test

Rats were tested in the rotarod apparatus (Multiserv, Lublin, Poland), similarly to the method described previously (Kotlinska et al. 2012). On the day preceding the experiment, all rats were accustomed to the apparatus (6 cm in diameter, 50 cm in length, subdivided into four areas by the disks, 25 cm in diameter, at a constant rotating speed of 9 rpm) in order to evaluate their performance. The time from the moment when the rod began to rotate until the rat fell off from it was measured (retention time). The rats that held onto the rotating rod for at least 2 min were selected for further experiments. In order to reveal the influence of acute ethanol or cholinesterase inhibitors on motor coordination, the latency of falling off the rotarod was determined 30 min after ethanol and 50 min after donepezil (1 or 3 mg/kg) or rivastigmine (0.5 or 1 mg/kg) administration. The animals that did not fall off the rotarod within 1 min were given the maximum score of 60 s.

#### *Experiment 1*

Effect of acute ethanol administration before the probe trial on spatial memory retrieval, locomotor activity, and motor coordination in rats

**Fig. 1:**
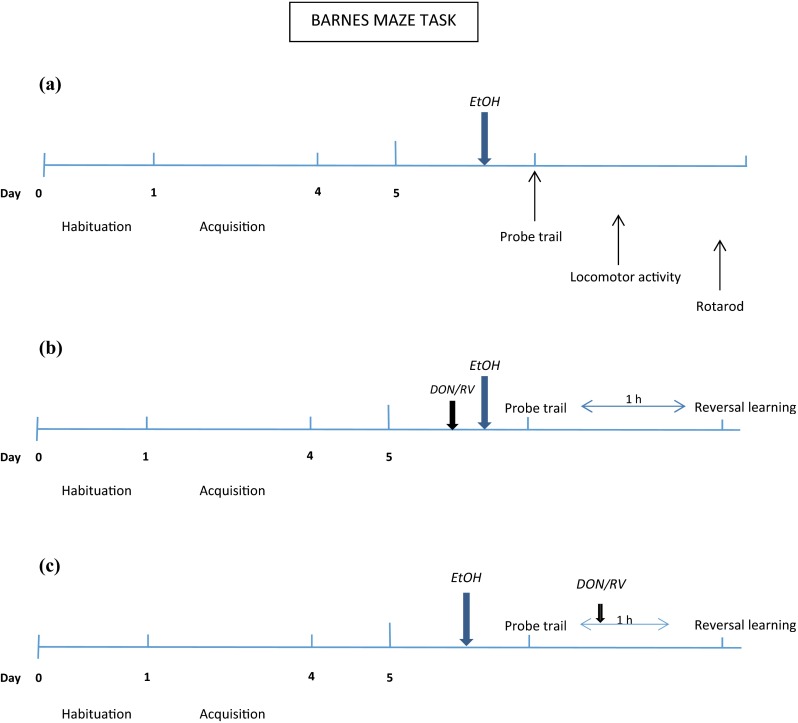
Diagram of the experimental design. **a** Effect of acute ethanol administration before the probe trial on spatial memory retrieval, locomotor activity, and motor coordination in rats. **b** Effect of donepezil/rivastigmine, given before the probe trial on spatial memory retrieval and cognitive flexibility, impaired by acute ethanol administration in Barnes maze task. **c** Effects of cholinesterase inhibitors, given before the reversal learning, on cognitive flexibility, impaired by acute ethanol administration before the probe trial in the Barnes maze task

The animals (*n* = 28) were trained in the Barnes maze task for 4 consecutive days with one session per day. Such sessions consisted of three trials separated by a 10-min inter-trial interval (acquisition phase), as described above (Fig. 1a). The animals were then randomly assigned to the one of four treatment groups (*n* = 7 per group) and subjected to the probe trial on the 5th day, 24 h after the last acquisition trials. One group received ethanol at the dose of 1.5 g/kg, the second group received 1.75 g/kg ethanol, the third group received 2.0 g/kg ethanol, and the fourth group received saline. Ethanol (10 % *w*/*v*) and saline were administered 30 min before the probe trial on the test day. Immediately after the probe trial, the influence of ethanol (1.5–2.0 g/kg, i.p.) on locomotor activity (locomotor activity cages) and the motor coordination (rotarod test) was assessed to exclude the influence of these ethanol doses on locomotor disturbances so as to exclude nonspecific effects in the memory task. On the basis of these experiments, the dose of ethanol of 1.75 g/kg was selected for further studies.

#### *Experiment 2*

Effect of donepezil, given before the probe trial, on spatial memory retrieval and cognitive flexibility impaired by acute ethanol administration in the Barnes maze task

During the acquisition phase, rats received three trials per day for 4 consecutive days (Fig. 1b). The probe trial was given on the 5th day, 24 h after the last acquisition trial. Before the probe trial, the animals (*n* = 82) were placed into six groups: saline + saline; saline + ethanol (1.75 g/kg); donepezil (1 mg/kg) + saline; donepezil (3 mg/kg) + saline; donepezil (1 mg/kg) + ethanol (1.75 g/kg); and donepezil (3 mg/kg) + ethanol (1.75 g/kg) (*n* = 13–14 per group). The doses of the donepezil (Gawel et al. 2014) and the dose of ethanol (experiment 1) were chosen based on our previous experiments. Fifty minutes and 30 min prior to the probe trial, the rats received donepezil and ethanol (1.75 g/kg) or saline, respectively, whereas the control group received saline instead of either the cholinesterase inhibitors or ethanol. One hour after the completion of the probe trial, three 180-s reversal learning trials were conducted, according to the procedure described above.

#### *Experiment 3*

Effect of rivastigmine, given before the probe trial on spatial memory retrieval and cognitive flexibility impaired by acute ethanol administration in the Barnes maze task

Rats were trained in the Barnes maze for 4 consecutive days (acquisition phase), with three trials per day (Fig. 1b). The probe trial was given on the 5th day, 24 h after the last acquisition trial. Before the probe trial, the animals (*n* = 72) were placed into six groups: saline + saline; saline + ethanol (1.75 g/kg); rivastigmine (0.5 mg/kg) + saline; rivastigmine (1 mg/kg) + saline; rivastigmine (0.5 mg/kg) + ethanol (1.75 g/kg); and rivastigmine (1 mg/kg) + ethanol (1.75 g/kg) (*n* = 12 per group). The doses of rivastigmine (Gawel et al. 2014) were chosen based on our previous experiment. Fifty minutes and 30 min prior to the probe trial, the rats received rivastigmine and ethanol (1.75 g/kg) or saline, respectively, whereas the control group received saline instead of either the cholinesterase inhibitors or ethanol. One hour after the completion of the probe trial, three 180-s reversal learning trials were conducted, according to the procedure described above.

#### *Experiment 4*

Effects of cholinesterase inhibitors, given before the reversal learning, on cognitive flexibility impaired by acute ethanol administration before the probe trial in the Barnes maze task

During the acquisition phase, the rats were trained in the Barnes maze for 4 days, with three trials per day, as described above (Fig. 1c). Then, on the 5th day, the animals (*n* = 72) were randomly placed into six groups: saline + saline; ethanol (1.75 g/kg) + saline; ethanol (1.75 g/kg) + donepezil (1 mg/kg); ethanol (1.75 g/kg) + donepezil (3 mg/kg); ethanol (1.75 g/kg) + rivastigmine (0.5 mg/kg); and ethanol (1.75 g/kg) + rivastigmine (1 mg/kg) (*n* = 12 per group). Ethanol was given 30 min before the probe trial, while donepezil or rivastigmine was given 10 min after the probe trial (50 min before the reversal learning trials).

#### *Experiment 5*

Effect of acute donepezil and rivastigmine on locomotor activity and motor coordination in rats

**Fig. 2:**
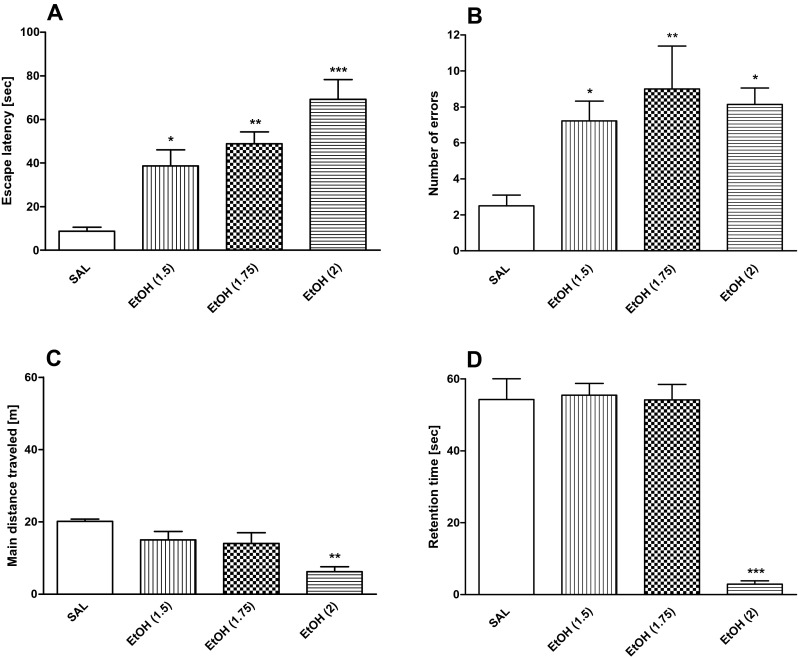
The influence of different doses of ethanol (1.5, 1.75, or 2 g/kg, i.p.) on **a** primary latency; **b** number of errors measured during the probe trial in the Barnes maze task; **c** locomotor activity; **d** motor coordination. Ethanol was given 30 min before all experiments. Results are expressed as mean ± SEM. ***P < 0.001, **P < 0.01, *P < 0.05 vs. saline-treated rats; (*n* = 7/group); *EtOH* ethanol, *SAL* saline

**Fig. 3:**
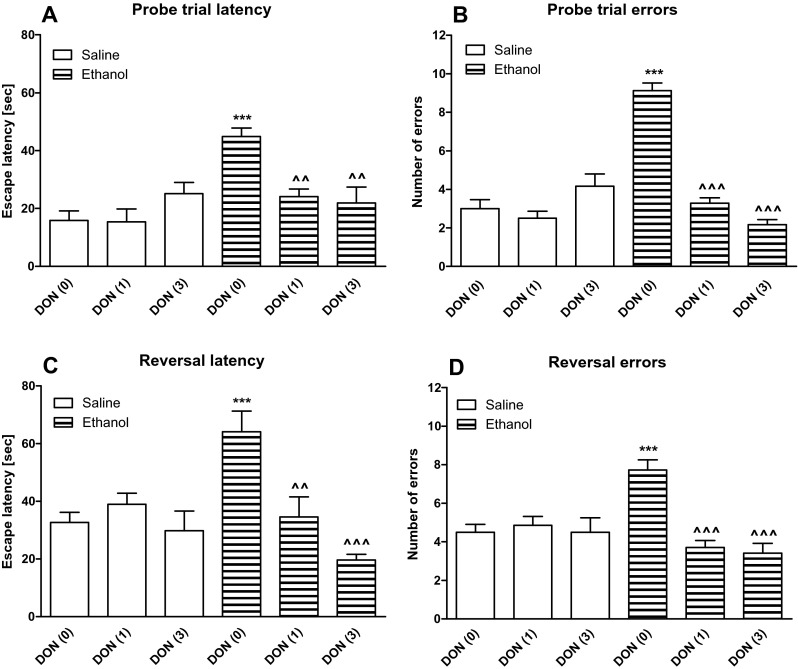
The influence of donepezil (1 or 3 mg/kg, i.p.) on the spatial memory impairments caused by acute ethanol (1.75 g/kg, i.p.) administration measured in the Barnes maze task in rats. Fifty minutes prior the probe trial, rats received donepezil, followed by ethanol (1.75 g/kg) or saline 20 min later (30 min before the probe trial). Primary latency (panels **a**, **c**) and number of errors (panels **b**, **d**), measured during the probe trial and reversal learning are shown as mean ± SEM of the three trials (*n* = 13–14/group). ***P < 0.001 vs. saline-treated rats; ^^^^ *P* < 0.01; ^^^^^ *P* < 0.001 vs. ethanol-treated rats. *DON* donepezil

The new cohort of animals (*n* = 38) was placed into five groups: saline, donepezil (1 mg/kg), donepezil (3 mg/kg), rivastigmine (0.5 mg/kg), and rivastigmine (1 mg/kg) (*n* = 7 per group). Fifty minutes after saline/donepezil/rivastigmine administration, the locomotor activity test, followed by the rotarod test, was performed (Figs. 2 and 3).

### Statistical analysis

**Fig. 4:**
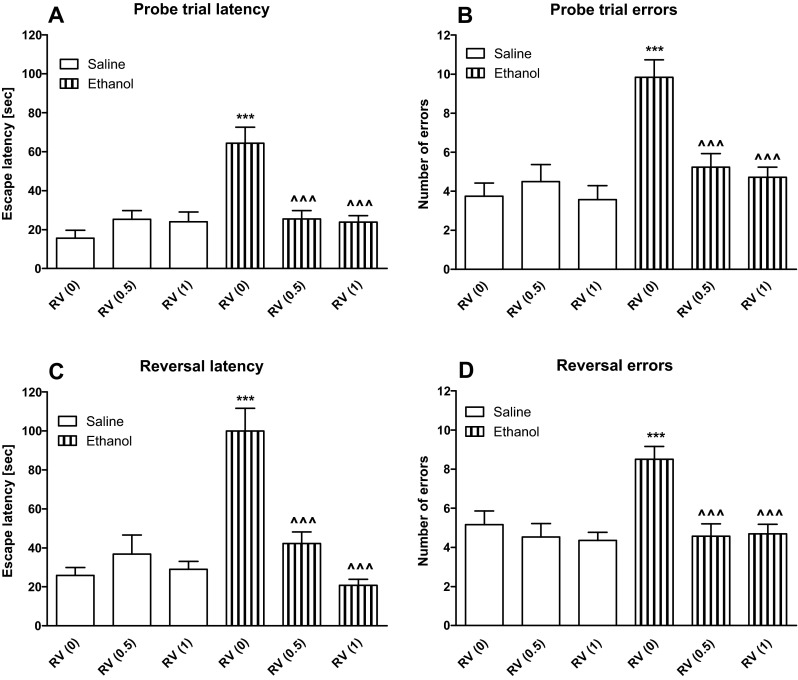
The influence of rivastigmine (0.5 or 1 mg/kg, i.p.) on the spatial memory impairments caused by acute ethanol (1.75 g/kg, i.p.) administration measured during the Barnes maze task in rats. Fifty minutes prior the probe trial, rats received rivastigmine followed by ethanol (1.75 g/kg) or saline 20 min later (30 min before the probe trial). Escape latency (panels **a**, **c**) and number of errors (panels **b**, **d**) measured at the probe trial and reversal learning are shown as mean ± SEM of the three trials (*n* = 12 group). **P < 0.01; ***P < 0.001 vs. saline-treated rats; ^^^^^ *P* < 0.001 vs. ethanol-treated rats. *RV* rivastigmine

Data from experiments 1, 4, and 5 were statistically analyzed by one-way analysis of variance (ANOVA), followed by the Tukey-Kramer post hoc test (Figs. 1 and 4). Data from experiments 2 and 3 were analyzed using two-way analysis of variance (ANOVA) with the factors “treatment” (between subjects) and “dose of donepezil/rivastigmine” (within subjects), followed by the Bonferroni post hoc test (Figs. 2 and 3). Results were presented as mean ± standard errors (SEM). *P* value less than 0.5 was considered statistically significant for all tests.

## Results

### *Experiment 1*

Effect of acute ethanol (1.5, 1.75, 2 g/kg) administration before the probe trial on spatial memory retrieval in the Barnes maze, locomotor activity, and motor coordination in rats

On the last day of acquisition (4th day, 1 day before randomization), the average latency (basal primary latency) in rats to reach the escape box (mean of three trials ± SEM) was 15.64 s ± 1.56 s.

On day 5, animals’ spatial memory was evaluated by the probe trial. A one-way ANOVA showed significant treatment effect on the primary latency [F(3,25) = 13.84; *P* < 0.001, Fig. 2a] and number of errors [F(3, 25) = 5.94; *P* < 0.01, Fig. 2b]. Unlike controls, ethanol-treated rats required more time (primary latency) to find the escape box at the doses of 1.5 (*P* < 0.05), 1.75 (*P* < 0.01), or 2 g/kg (*P* < 0.001) and showed a statistically significant increase in the number of primary errors to reach the escape box at the doses of 1.5 (*P* < 0.05), 1.75 (*P* < 0.01), or 2 g/kg (*P* < 0.05). A one-way ANOVA showed that there was significant effect of the treatment on the locomotor activity [F(3,25) = 6.08, *P* < 0.05, Fig 2c] and coordination [F(3,25) = 48.45, *P* < 0.001, Fig. 2d]. Post hoc (Tukey-Kramer) test indicated that ethanol at the dose of 2 g/kg significantly decreased locomotor activity (*P* < 0.01) and coordination (*P* < 0.001). Thus, the results of these experiments have shown that ethanol at the doses of 1.5 and 1.75 g/kg induced memory impairment in the Barnes maze task without affecting locomotor activity and motor coordination. Because our unpublished data indicated that the ethanol dose of 1.5 g/kg affected only one parameter (number of primary errors) tested in the Barnes maze, we decided to apply a higher dose of ethanol (1.75 g/kg).

### *Experiment 2*

Effect of donepezil, given before the probe trial on spatial memory retrieval and cognitive flexibility impaired by acute ethanol administration in the Barnes maze task

On the last day of acquisition (4th day, 1 day before randomization), the average latency (basal primary latency) in rats to reach the escape box (mean of three trials ± SEM) was 21.31 s ± 1.27 s.

During the probe trial on day 5, a two-way ANOVA showed statistically significant differences between the groups in the primary latency [treatment F(1,76) = 9.77, *P* < 0.01; dose of donepezil F(2,76) = 5.81, *P* < 0.01; treatment × dose of donepezil interaction F(2,76) = 14.96, *P* < 0.001; Fig. 3a] and a number of primary errors [treatment F(1,76) = 25.94, *P* < 0.001; dose of donepezil F(2,76) = 36.31, *P* < 0.001; treatment × dose of donepezil interaction F(2,76) = 51.61, *P* < 0.001; Fig. 3b]. Moreover, post hoc (Bonferroni) test revealed that ethanol-treated rats showed statistically significant increase in the primary latency (*P* < 0.001) and errors (*P* < 0.001) committed to reach the escape box, as compared to control animals. Donepezil, given before ethanol in the probe trial, prevented/attenuated the short-time spatial memory impairment induced by ethanol. Donepezil, at both doses (1 or 3 mg/kg, i.p.), decreased the primary latency (*P* < 0.01) and the number of primary errors (*P* < 0.001) in the ethanol-treated rats.

Reversal learning trials were performed 1 h after the probe trial. A two-way ANOVA showed significant effect of a dose of donepezil [F(2,76) = 11.74, *P* < 0.001] and treatment × dose of donepezil interaction [F(2,76) = 10.97, *P* < 0.001; Fig. 3c]. Moreover, a two-way ANOVA indicated statistically significant differences between the groups in the number of primary errors [treatment F(1,76) = 6.40, *P* < 0.05; dose of donepezil F(2,76) = 10.17, *P* < 0.001; treatment × dose of donepezil interaction F(2,76) = 9.48, *P* < 0.001; Fig. 3d]. Post hoc (Bonferroni) test revealed a significant increase in the primary latency (*P* < 0.001) and the number of primary errors (*P* < 0.001) committed to reach the target hole in the ethanol-treated group. Pretreatment with donepezil prevented the cognitive flexibility impairment induced by ethanol. Donepezil at the dose of 1 mg/kg (*P* < 0.01) or 3 mg/kg (*P* < 0.001) decreased primary latency and, at these doses, decreased a number of primary errors (*P* < 0.001) committed to reaching the escape box in the ethanol-treated rats.

### *Experiment 3*

Effect of rivastigmine, given before the probe trial on spatial memory retrieval and cognitive flexibility impaired by acute ethanol administration in the Barnes maze task

On the last day of acquisition (4th day, 1 day before randomization of animals), the average latency (basal primary latency) to reach the escape box (mean of three trials ± SEM) was 18.32 s ± 0.98 s.

During the probe trial on day 5, a two-way ANOVA showed statistically significant differences between the groups in the primary latency [treatment F(1,66) = 16.96, *P* < 0.001; dose of rivastigmine F(2,66) = 4.74, *P* < 0.05; treatment × dose of rivastigmine interaction F(2,66) = 16.00, *P* < 0.001; Fig. 4a] and the number of primary errors [treatment F(1,66) = 19.75, *P* < 0.001; dose of rivastigmine F(2,66) = 7.78, *P* < 0.01; treatment × dose of rivastigmine interaction F(2,66) = 9.57, *P* < 0.001; Fig. 4b]. Post hoc (Bonferroni) test indicated a significant increase in the primary latency (*P* < 0.001) and the number of primary errors (*P* < 0.001) to reach the escape box in the ethanol-treated group. Rivastigmine, given before ethanol in the probe trial, prevented the short-time memory impairment induced by ethanol. Rivastigmine at both doses (0.5 or 1 mg/kg, i.p.) decreased the primary latency (*P* < 0.001) and the number of primary errors (*P* < 0.001) committed to reaching the escape box, in the ethanol-treated rats.

Reversal learning trials were performed 1 h after the probe trial. A two-way ANOVA showed statistically significant differences between the groups in the primary latency [treatment F(1,66) = 13.60, *P* < 0.001; dose of rivastigmine F(2,66) = 11.99, *P* < 0.001; treatment × dose of rivastigmine interaction F(2,66) = 24.40, *P* < 0.001; Fig. 4c] and the number of primary errors [treatment F(1,66) = 8.35, *P* < 0.01; dose of rivastigmine F(2,66) = 9.90, *P* < 0.001; treatment × dose of rivastigmine interaction F(2,66) = 3.60, *P* < 0.05; Fig. 4d]. Post hoc (Bonferroni) test revealed an increase in the primary latency (*P* < 0.001) and a higher number of errors committed (*P* < 0.001) to reaching the escape box, in the ethanol-treated group. Pretreatment with rivastigmine prevented the cognitive flexibility impairment induced by ethanol. Rivastigmine, given prior to ethanol at both doses (1 and 3 mg/kg), decreased the primary latency (*P* < 0.001) and the number of primary errors (*P* < 0.001) to reaching the escape box.

### *Experiment 4*

The influence of cholinesterase inhibitors, given before the reversal learning on cognitive flexibility impaired by ethanol administration before the probe trial in the Barnes maze task

On the last day of acquisition (4th day, 1 day before randomization of animals), the average latency (basal primary latency) in rats to reaching the escape box (mean of three trials ± SEM) was 20.99 ± 0.95 s.

**Fig. 5:**
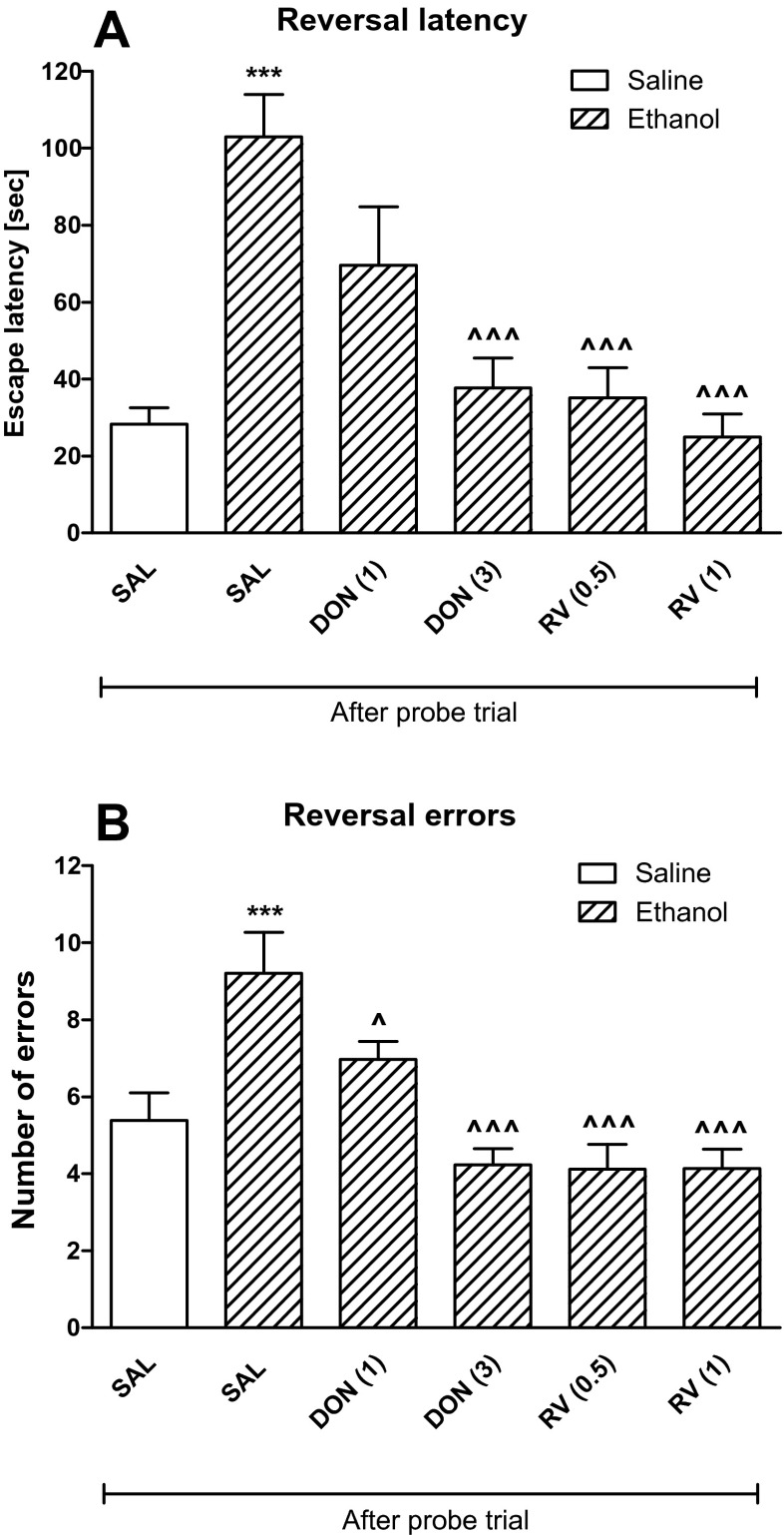
The influence of donepezil (1 or 3 mg/kg, i.p.) and rivastigmine (0.5 or 1 mg/kg, i.p.) on the ethanol-impaired cognitive flexibility in the reversal learning during the Barnes maze task in rats. Ethanol (1.75 g/kg, i.p.) was given 30 min before the probe trial, while donepezil or rivastigmine were given 10 min after the probe trial (50 min before the reversal learning trials). Primary latency (panel **a**) and number of errors (panels **b**) measured during the reversal learning are shown as mean ± SEM of the three trials (*n* = 12/group). ***P < 0.001 vs. SAL/SAL group of rats; ^^^*P* < 0.001 vs. EtOH/SAL group of rats

In the probe trial (5th day), a one-way ANOVA [escape latency F(5,66) = 6.37, *P* < 0.001, Fig. 5a; number of errors F(5,66) = 6.33, *P* < 0.001, Fig. 5b] indicated that, unlike control animals, ethanol-treated animals failed to learn a new location (escape latency *P* < 0.001; number of errors *P* < 0.001) during the reversal phase, suggesting a loss of cognitive flexibility. Both cholinesterase inhibitors attenuated cognitive flexibility impairment induced by ethanol. Thus, donepezil given before the reversal learning in the ethanol-treated rats, only at the dose of 3 mg/kg, decreased primary latency (*P* < 0.001) and the number of errors (*P* < 0.001). Both doses of rivastigmine (0.5 or 1 mg/kg) given before the reversal learning in the ethanol-treated rats decreased primary latency and the number of errors (*P* < 0.001).

### *Experiment 5*

Effect of donepezil and rivastigmine on locomotor activity and rotarod

A one-way ANOVA revealed that acute administration of donepezil and rivastigmine alone disturbed neither locomotor activity [F(4,33) = 1.02, *P* > 0.05] nor motor coordination [F(4,33) = 0.42, *P* > 0.05] in rats (data not shown).

## Discussion

The present study has shown that acute ethanol administration before the probe trial (24 h after the last acquisition trial) impaired spatial memory and cognitive flexibility in the Barnes maze task. These ethanol effects were prevented by pretreatment with the cholinesterase inhibitors, donepezil and rivastigmine, given before ethanol in the probe trial. Moreover, these cholinesterase inhibitors given before the reversal learning trials were capable of reversing the cognitive flexibility impairments observed after ethanol administration (before the probe trial) in this maze. Although we have not observed differences in effectiveness of both cholinesterase inhibitors in preventing ethanol-induced cognitive impairments when these drugs were given before probe trial, rivastigmine was more effective than donepezil when it was given before the reversal learning, most probably due to its broader inhibitory spectrum. Furthermore, both cholinesterase inhibitors, given alone, did not affect locomotor activity and motor coordination at the doses used in the Barnes maze task.

The reduction in hippocampal ACh levels specifically correlates with impairments in the spatial memory (Ikegami 1994; Mishima et al. 2000; Gold 2003). Ethanol decreases ACh release in the hippocampus (Henn et al. 1998) and may impair spatial memory. The results of the present experiments show that acute ethanol administration impaired the use of a previously learned spatial reference memory task. In our study, animals were first trained to navigate to the escape shelter in the Barnes maze for 4 days and then (day 5) were tested under one of three doses of ethanol (saline control, 1.5, 1.75, or 2.0 g/kg i.p. injection) for 30 min, following ethanol administration. Ethanol produced a significant dose-dependent impairment in the previously learned spatial information. Thus, pretest administration of ethanol impaired the retrieval of spatial memory on the test day, compared to saline-treated animals. Furthermore, our experiments indicated that the ethanol dose of 1.75 g/kg was “optimal” for the future study, because it produced memory impairment in the Barnes maze task without affecting locomotor activity and motor coordination. The impairment of spatial memory was confirmed by earlier reports. In these, acute ethanol administration before the task on the test day impaired spatial learning and memory in the water/radial maze in animals (Matthews et al. 1995; White et al. 1998; Berry and Matthews 2004).

Systemic (oral) administration of the cholinesterase inhibitors, donepezil and rivastigmine, is known to increase extracellular ACh concentration in the hippocampus and prefrontal cortex (Liang and Tang 2006; Kosasa et al. 1999). In the present study, systemic (i.p.) preadministration of donepezil and rivastigmine before ethanol prevented ethanol-induced spatial memory deficits in the Barnes maze task. These data may suggest that the cholinergic system is closely associated with the spatial memory impairment that is induced by acute ethanol administration. This is in agreement with several investigations concerning memory, indicating an interaction between ethanol and the cholinergic system in the laboratory animals (Arendt 1994; Pick et al. 1993; Rezayof et al. 2008).

Reversal learning is a type of discrimination learning that measures cognitive flexibility (Stalnaker et al. 2009), and impairment of the reversal learning has been interpreted as perseveration of behavior (Obernier et al. 2002). Previous experiments have indicated that reversal learning is impaired after acute ethanol in nonhuman primates (Jedema et al. 2011) and after ethanol binge in rats (Obernier et al. 2002; Kuzmin et al. 2012). Our data extend these observations and show, for the first time, that acute ethanol (1.75 g/kg) administration impaired reversal learning in the Barnes maze task in rats. Previous studies have also established that chronic ethanol abuse impairs reversal performance and reduces orbitofrontal cortex activity (Volkow et al. 1993; Volkow and Fowler 2000; Fortier et al. 2008). Such effect of chronic ethanol abuse has been correlated with a higher incidence of relapse (Noël et al. 2002). In turn, acute effect of ethanol on the orbitofrontal cortex may contribute to the impaired inhibition of prepotent responding and increased impulsivity that presumably underlie poor decisions associated with subintoxicating levels of ethanol (de Wit et al. 2000; Dougherty et al. 2008). Our study revealed that cholinesterase inhibitors given either before ethanol administration prior to probe trial or before reversal learning trials improved reversal learning processes. Furthermore, rivastigmine at both doses (0.5 and 1 mg/kg, i.p.), but donepezil only at a higher dose (3 mg/kg, i.p.), given prior to the reversal learning, reversed the ethanol-induced impairment in cognitive flexibility. Thus, rivastigmine appears to exert a more beneficial effect than donepezil in reversing ethanol-induced cognitive impairments, probably due to its wider spectrum of inhibitory activity. These data indicate the valuable effect of cholinesterase inhibitors in cognitive disturbance induced by acute ethanol administration.

Although ACh inhibitors counteracted ethanol-induced impairment in spatial memory and cognition, the precise mechanism of this phenomenon is not completely understood. Ethanol is known to alter the activity of multiple signaling molecules involved in synaptic processing in the brain (Nevo and Hamon 1995; Diamond and Gordon 1997), including activation of ɣ-aminobutyric acid-A (GABA-A) receptors or blockade of N-methyl-d-aspartate (NMDA) glutamate receptors (Lovinger et al. 1990; Göthert and Fink 1989). Moreover, ethanol effects on glutamate and GABA-A receptors contribute to the ethanol-induced changes in long-term potentiation (LTP) and long-term depression (LTD), two forms of synaptic plasticity thought to underlie memory acquisition (Zorumski et al. 2014). It has been indicated that activation of GABAergic neurons negatively modulates cholinergic cell bodies in the septum (Imperato et al. 1993; Giovannini et al. 1994), but activation of NMDA receptors either stimulates or inhibits hippocampal ACh release (Giovannini et al. 1998; Moor et al. 1996). Furthermore, glutamatergic activation of the PFC by a direct input from the hippocampus seems to be an essential part of the mechanisms of spatial memory (Vickery et al. 1997; Lee and Kesner 2003). Studies in rodents indicated that cholinergic stimulation facilitates the processing of incoming information. Application of donepezil or rivastigmine generally elevates ACh levels and thus affects both nicotinic (nAChRs) and muscarinic (mAChRs) cholinergic receptors. In addition, activation of nAChRs enhances excitatory input to the hippocampal area CA3 from the entorhinal cortex (Giocomo and Hasselmo 2005) and from dentate gyrus (Radcliffe et al. 1999); thus, this may enhance the efficiency of encoding spatial information. In our study, both cholinesterase inhibitors facilitated acquisition of new information in the reversal phase of the Barnes maze task.

There are few studies that have investigated the role of the cholinergic system in memory retrieval (Zarrindast et al. 1996; Martí Barros et al. 2004; Soares et al. 2006; Piri and Zarrindast 2011). Our experiments extend these data and indicate that pretreatment with cholinesterase inhibitors reduced the ethanol-induced impairment in retrieval of spatial memory. Such outcome suggests that ACh is necessary for recalling the well-trained spatial memory disturbed by ethanol. It has also been implied that well-trained memory (experimentally experienced) is stored in the neocortex and retrieved by glutamatergic top-down signals from the PFC (Hasegawa et al. 1998). Activation of mAChRs but not nAChRs in the cortical regions is involved in this mechanism (Saper 1984). This allows retrieving memory by regulating the dynamics of the neuronal network in the glutamatergic system. The spatial memory deficits induced by ethanol are correlated with the decrease in glutamate output and might be accompanied by the reduction in ACh transmission mediated by septal GABA receptors in the hippocampus (Shimizu et al. 1998). Because there is an interaction between PFC and hippocampus in controlling memory retrieval (Preston and Eichenbaum 2013), prior administration of cholinesterase inhibitors to alcohol intake may increase ACh level and prevent reduction in glutamate output induced by ethanol. It seems that such mechanism may be involved in the effects produced by donepezil and rivastigmine on the impaired memory retrieval induced by acute ethanol administration.

In conclusion, our study indicated, for the first time, that cholinesterase inhibitors, donepezil and rivastigmine, prevent impairment of short-term spatial memory and memory flexibility induced by acute ethanol given after the acquisition phase in the Barnes maze task. Furthermore, both drugs (donepezil only at higher dose) prevent the cognitive flexibility impairment induced by acute ethanol when given prior to the reversal learning of the task. Thus, our data show that not only is ACh beneficial in memory retrieval, following acute ethanol impairment, but also is necessary for the improvement of learning and memory disturbed by ethanol. Further studies are required to elucidate the molecular mechanisms involved in these effects.
